# Triblock Copolymer Micelles with Tunable Surface Charge as Drug Nanocarriers: Synthesis and Physico-Chemical Characterization

**DOI:** 10.3390/nano12030434

**Published:** 2022-01-27

**Authors:** Radostina Kalinova, Ivaylo Dimitrov

**Affiliations:** Institute of Polymers, Bulgarian Academy of Sciences, Akad. G. Bonchev St., bl. 103-A, 1113 Sofia, Bulgaria; kalinova@polymer.bas.bg

**Keywords:** amphiphilic block copolymers, synthesis, micelles, stability, curcumin, drug delivery, nanocarriers

## Abstract

Polymeric micelles have gained increasing interest as efficient drug delivery systems for cancer treatment and diagnosis. The aim of the present study was to construct and to evaluate novel polymeric nanosized drug carriers with tunable surface charges. Initially, amphiphilic triblock copolymers with predetermined molar mass characteristics were synthesized by applying controlled polymerization techniques. The copolymers self-assembled in aqueous media into core–shell spherical micelles, comprising a biodegradable hydrophobic poly(D,L-lactide) core, positively charged middle layer of poly((2-dimethylamino)ethyl methacrylate), and an outer shell of neutral hydrophilic poly(oligo(ethylene glycol) methyl ether methacrylate), with various densities of the short polyether side chains. The block copolymer micelles with average diameters of about 70 nm and surface charges varying from strongly positive to neutral were characterized and loaded with the model, natural, hydrophobic drug curcumin. Characteristics such as drug loading efficiency, in-vitro drug release profiles, and stability under physiological conditions were evaluated and discussed in terms of nanocarriers’ composition. As a result, the most promising candidates for potential application in nanomedicine were identified.

## 1. Introduction

Bioavailability is a key pharmacokinetic property of drugs defining the part of initially introduced amount of active drug which is available for systemic circulation and subsequently reaches the target site of action [1]. Drugs characterized with a poor bioavailability are not able to reach the concentration required to reveal their pharmacological action. Low drug bioavailability usually is due to poor aqueous solubility, since more than 70% of newly developed drug candidates are hydrophobic in nature [2]. Other factors affecting drug bioavailability are inappropriate hydrophilic–lipophilic balance (HLB), metabolism of a drug before it reaches systemic circulation, and degradation in the gastrointestinal tract [3,4,5]. The increasing interest of applying nanotechnology in various biomedical applications, including in drug delivery, has led to improved drug efficacy by selecting appropriate nanocarriers for achieving a controlled release and higher bioavailability [6]. The optimal size range of nanoparticles used as drug carriers is usually within 10–200 nm [7]. Thus, they can selectively accumulate within the tumor site, penetrating the leaky vascular walls in cancer tissues via the so-called enhanced permeability and retention (EPR) effect [8,9]. The spontaneous accumulation and retention of the administrated drug nanocarriers into the solid tumors’ vicinity is usually associated with the term “passive targeting”. Historically, liposomes were among the first nanosized drug delivery systems studied [10]. Other classes of nanostructured materials used for drug delivery or imaging applications include polymeric (micelles, dendrimers, or polymersomes) and inorganic (quantum dots, silica, or metal) nanoparticles [11]. Polymeric micelles formed via self-assembly of amphiphilic macromolecules in aqueous media have numerous features making them attractive for drug delivery. These include biodegradability, controlled or sustained release, biocompatibility with tissues and cells, and tunable particle size [12]. If appropriately chosen, they could be relatively nontoxic, nonimmunogenic, and stable in the blood stream [13]. Thus, a number of biopolymers such as chitosan, cellulose, collagen, albumin, alginate, and gelatin have been utilized in drug delivery systems due to their inherent biocompatibility and biodegradability [14]. Synthetic polymers, on the other hand, offer great possibilities for fine tuning the nanocarriers’ properties, depending on the specific application. With the development of controlled polymerization and modification methods, a precise control over the copolymer composition, dispersity, functionality, hydrophilic–hydrophobic balance, and pharmacokinetics of the nanocarriers could be achieved [15,16,17]. The polymer micelles are usually characterized as core–shell structures with hydrophobic cores comprising biodegradable macromolecules that are capable of drug accommodation and determining the stability and release properties. Typically, the core forming hydrophobic polymers are polyesters such as poly(lactic acid) (PLA), poly(glycolic acid) (PGA), poly(lactic-*co*-glycolic acid) (PLGA), polycaprolactone (PCL), or various poly(amino acid)s [18,19]. The hydrophilic surface (“corona”) of self-assembled amphiphilic copolymer nanostructures, usually comprising poly(ethylene glycol) (PEG), imparts the so called “stealth” effect [20]. As a result, a prolonged nanocarriers’ blood circulation is achieved due to the minimized risk of their opsonization followed by reticuloendothelial system uptake [21]. Although PEG is one of the most widely used corona-forming hydrophilic synthetic polymers due to its biocompatibility and antifouling properties, its presence on the nanocarrier surface could also result in reduced cellular uptake and endosomal escape [22]. Therefore, various neutral or charged hydrophilic polymers such as poly((2-dimethylamino)ethyl methacrylate) (PDMAEMA), poly(*N*-vinyl-2-pyrrolidone) (PVP), poly(2-hydroxyethyl methacrylate) (PHEMA), poly(2-oxazoline)s, or poly(acrylic acid) (PAA) have been explored as micelles’ corona forming blocks [23,24,25,26,27]. A positively charged surface of the nanocarriers is beneficial for their enhanced cellular uptake through the strong interaction with the negatively charged cell membrane [28]. On the other hand, there is an increased possibility of surface charged particles’ rapid elimination from the blood stream before reaching the target cells through protein adsorption and rapid clearance by the mononuclear phagocytic system [29].

Curcumin (Curc) is a hydrophobic polyphenolic compound (diferuloylmethane) isolated from the roots of the plant *Curcuma longa*. It has gained significant popularity due to its numerous biological activities [30,31,32]. As a result of its anticancer, antioxidant, antidiabetic, anti-inflammatory, and other properties, Curc has been considered as a potential multipurpose drug. In attempts to overcome the drug’s inherent poor aqueous solubility, photodegradability, and chemical instability at physiological conditions Curc has been incorporated into polymer carriers such as complex microparticles [33], nanocapsules [34], and nanospheres [35]. However, in most cases, the drug was encapsulated into the hydrophobic core of various amphiphilic diblock or triblock copolymer micelles, characterized either with neutral (mostly PEG) [36,37,38,39,40,41,42] or positively charged corona [43,44,45,46].

The current work deals with the design and preparation of new triblock copolymer micelles with tunable surface charge comprising a biodegradable hydrophobic block of poly(D,L-lactide), positively charged central PDMAEMA-block and a neutral hydrophilic block of poly(oligo(ethylene glycol) methyl ether methacrylate) (POEGMA). The applied controlled polymerization methods allowed us to finely tune the copolymer’s composition. The copolymers obtained self-assembled in aqueous media, forming stable micelles with sizes in the 60–70 nm range and surface charge from strongly positive to neutral depending on the POEGMA content. The copolymer micelles were physico-chemically characterized and loaded with the model drug curcumin. Characteristics such as drug loading efficiency, drug loading capacity, and drug release profiles were evaluated in the light of potential application of the triblock copolymer micelles as nanocarriers in drug delivery systems. Overall, the main advantage of the presented amphiphilic triblock copolymers with tunable length of the POEGMA blocks is in the feasible control over the formed micelles’ surface charge, which would be much more difficult to achieve from a synthetic point of view if PEG was used as a hydrophilic neutral block.

## 2. Materials and Methods

### 2.1. Materials

All reagents and other substances used in the study were obtained from Sigma-Aldrich (St. Louis, MO, USA). D,L-lactide (LA, 99%) was purified through recrystallization from 95:5 *v*/*v* toluene/ethyl acetate. Oligo (ethylene glycol) methyl ether methacrylate (OEGMA, *M*_n_ ~ 500 Da) was passed under an argon pressure firstly through a column packed with a neutral Al_2_O_3_ and then through a column packed with a basic Al_2_O_3_ in order to remove the inhibitors. *N*,*N*-Dimethylaminoethyl methacrylate (DMAEMA, 98%) was purified by passing it through a column packed with Al_2_O_3_. The phenethyl alcohol (PEA, ≥99%) initiator was purified by vacuum distillation just before use. Triethylamine (TEA, 99%) was distilled and kept over KOH. The solvents dichloromethane (DCM, 99.5%), tetrahydrofuran (THF, ≥99%), toluene (98%), and anisole (≥99%) were purified by applying standard procedures. The catalyst tin(II) 2-ethylhexanoate (Sn(Oct)_2_, 92.5–100%), the initiator 2-bromo-2-methylpropionyl bromide (BIBB, 98%), 1,1,4,7,10,10-hexamethyltriethylenetetramine (HMTETA, 97%), (*N*,*N*,*N*′,*N*″,*N″*-pentamethyldiethylenetriamine (PMDETA, 99%), copper(I) bromide (CuBr, 99.999%), copper(I) chloride (CuCl, 99.999%), and the model drug curcumin were used without further purification.

### 2.2. Synthesis of the Hydrophobic Polyester Block (PLA) 

The modified literature procedure was performed as follows [47]: LA (2 g, 13.9 mmol) was placed into a Schlenk tube, dried in vacuum for 1 h, and dissolved in 7 mL of toluene under an inert atmosphere upon slight heating. The PEA initiator (0.055 mL, 0.467 mmol) and Sn(Oct)_2_ (0.06 mL, 0.185 mmol) solution in 1 mL of toluene were added. The polymerization proceeded at 90 °C for 24 h. The solvent was removed, the crude product was redissolved in DCM, poly(D,L-lactide) was precipitated in chilled methanol and dried under reduced pressure. Yield: 1.88 g (94%). ^1^H NMR (600 MHz, CDCl_3_, *δ*, ppm): 7.30–7.15 (Ar, C_6_*H*_5_), 5.21–5.15 (C*H*-(CH_3_)-O), 4.36 (C*H*-(CH_3_)-OH + Ar-C*H*_2_-CH_2_-O), 2.94 (Ar-CH_2_-C*H*_2_-O), 1.55 (CH-(C*H*_3_)-O), 1.49 (CH-(C*H*_3_)-OH).

### 2.3. Conversion of PLA into a Macroinitiator (PLA-Br) 

TEA (0.177 mL, 1.27 mmol) was added to a PLA (1.5 g, 0.326 mmol) solution in DCM (20 mL). In the next step, BIBB (0.12 mL, 0.978 mmol) solution in DCM (5 mL) was slowly added to the reaction mixture via a dropping funnel following previously described procedure [48]. The reaction proceeded for 24 h at 25 °C. The reaction mixture was concentrated on a rotary evaporator to 1/3 of the initial volume and an activated charcoal (~10 mg) was added using the tip of a small spatula. The mixture was stirred overnight, filtrated and the macroinitiator was precipitated from the clear solution into cold methanol. Yield: 1.16 g (77%) ^1^H NMR (600 MHz, CDCl_3_, *δ*, ppm): 7.30–7.15 (Ar, C_6_*H*_5_), 5.21–5.15 (C*H*-(CH_3_)-O), 4.36 (C*H*-(CH_3_)-OH + Ar-C*H*_2_-CH_2_-O), 2.94 (Ar-CH_2_-C*H*_2_-O), 1.98 (C-(Br)-(C*H*_3_)_2_), 1.55 (CH-(C*H*_3_)-O).

### 2.4. Synthesis of Amphiphilic Diblock Copolymer (PLA-b-PDMAEMA) 

The second block was polymerized according to an already reported procedure [49]. The macroinitiator PLA-Br (0.5 g, 0.104 mmol) and CuBr (0.015 g, 0.104 mmol), were placed into a Schlenk tube, dried under vacuum for an hour and dissolved in 3 mL of THF. In the next step, DMAEMA (0.35 mL, 2.08 mmol) was added, and the reaction mixture was degassed by bubbling argon through a syringe for one hour. Finally, HMTETA (0.057 mL, 0.208 mmol) was injected into the reaction mixture via a degassed syringe. The polymerization proceeded for 5 h at 60 °C (oil bath) in an argon atmosphere. The reaction mixture was passed through a column containing neutral aluminum oxide in order to remove the catalytic complex. The clear solution was concentrated on a rotary evaporator and the product was precipitated in cold hexane. Yield: 0.66 g (80%) ^1^H NMR (600 MHz, CDCl_3_, *δ*, ppm): 7.30–7.12 (Ar, C_6_*H*_5_), 5.21–5.15 (C*H*-(CH_3_)-O), 4.34 (C*H*-(CH_3_)-OH + Ar-C*H*_2_-CH_2_-O), 4.08 (O-C*H*_2_-CH_2_-N), 2.94 (Ar-CH_2_-C*H*_2_-O), 2.62 (O-CH_2_-C*H*_2_-N), 2.32 (N-(C*H*_3_)_2_), 1.90–1.82 (C*H*_2_-C-(CH_3_)), 1.55 (CH-(C*H*_3_)-O), 1.05–0.90 (C-(C*H*_3_)_2_ + CH_2_-C-(C*H*_3_)).

### 2.5. Synthesis of Poly(D,L-Lactide)-b-Poly(N,N-Dimethylaminoethyl Methacrylate)-b-Poly(Oligo(Ethylene Glycol) Methyl Ether Methacrylate) (PLA-b-PDMAEMA-b-POEGMA) Amphiphilic Triblock Copolymer

The third block was polymerized by modifying a literature procedure [50]. Typically, CuCl (0.017 g, 0.075 mmol) was added to a solution of PLA-b-PDMAEMA (0.3 g, 0.0375 mmol) and OEGMA (0.375 g, 0.75 mmol) in anisole (2.5 mL). The oxygen was eliminated by degassing the mixture with an Ar for 30 min. Separately, a 0.15 M solution of PMDETA in anisole was prepared, degassed, and 0.5 mL were transferred via syringe into the reaction vessel. The combined solution was further degassed for another 30 min and immersed into a preheated to 60 °C oil bath. The polymerization was completed in 24 h. The solvent was removed on a rotary evaporator, the residue was redissolved in 15 mL of ethanol, and mixed with 50 mL of water. The mixture was subjected to ultrafiltration using a membrane with molecular weight cut-off 1000 Da. The purified triblock polymer was isolated from the solution through lyophilization. Yield: 0.44 g (65%) ^1^H NMR (600 MHz, CDCl_3_, *δ*, ppm): 7.30–7.18 (Ar, C_6_*H*_5_), 5.20–5.15 (C*H*-(CH_3_)-O), 4.35 (C*H*-(CH_3_)-OH + Ar-C*H*_2_-CH_2_-O), 4.25–4.07 (COO-C*H*_2_-CH_2_-O + O-C*H*_2_-CH_2_-N), 3.64 (O-C*H*_2_-C*H*_2_-O), 3.37 (-CH_2_-CH_2_-O-C*H*_3_), 2.94 (Ar-CH_2_-C*H*_2_-O), 2.65 (O-CH_2_-C*H*_2_-N), 2.36 (N-(C*H*_3_)_2_), 1.90–1.83 (C*H*_2_-C-(CH_3_)_PDMAEMA_ + C*H*_2_-C-(CH_3_)_POEGMA_), 1.56 (CH-(C*H*_3_)-O), 1.24-0.87 (CH_2_-C-(C*H*_3_)_POEGMA_ + C-(C*H*_3_)_2_ + CH_2_-C-(C*H*_3_)_PDMAEMA_).

### 2.6. Characterization

Proton nuclear magnetic resonance (^1^H NMR) analyses were performed using a Bruker Avance II+ 600 MHz spectrometer (Billerica, MA, USA). Size exclusion chromatography (SEC) was run on a Shimadzu Nexera XR (Kyoto, Japan) HPLC instrument in tetrahydrofuran at a flow rate of 1 mL min^−1^. The polymers’ molar-mass characteristics were determined using a RID-20A differential refractive index detector on the following set of columns: 10 μm PL gel mixed-B, 5 μm PL gel 500 Å, and 50 Å. Narrow dispersity polystyrene (PS) standards were applied for the instrument’s calibration and for the calculations. The ultraviolet-visible (UV/Vis) spectrophotometric analyses were performed using a DU 800 Beckman Coulter (Brea, CA, USA) instrument with a Peltier temperature controller. The infrared spectra were obtained from an IRAffinity-1 Fourier transform infrared (FTIR) spectrophotometer (Shimadzu, Kyoto, Japan). Transmission electron microscope (TEM) images were taken on a high-resolution scanning JEM-2100 analytical electron microscope (JEOL, Tokyo, Japan) with variable accelerating voltage (80–200 kV). The samples’ preparation was done by drop-casting of the micellar dispersions onto TEM grids, followed by solvent evaporation. The images were recorded using a charge-coupled device camera (GATAN Orius 832 SC1000, Pleasanton, CA, USA) and were processed via GATAN Microscopy Suite^®^ Software. The statistical analysis of the images obtained was completed using the ImageJ software. The dynamic light scattering (DLS) measurements were performed on a NanoBrook Plus PALS (Brookhaven Instruments, Holtsville, NY, USA) particle size and zeta (ζ) potential analyzer. The instrument operated at λ = 660 nm and 90 degrees scattering angle via the incorporated high power 35 mW diode laser. The average hydrodynamic diameters (*d_H_*) of the block copolymer micelles were obtained by applying the Stokes-Einstein equation:

*d_H_* = *kT*/(3*πηD*) (1)

(*k*—Boltzmann’s constant; *T*—temperature (K), *η*—viscosity, *D*—diffusion coefficient).

The phase analysis light scattering was utilized to determine the electrophoretic mobility of the surface charged micelles’ dispersions. Thus, the particles’ *ζ*-potentials were derived by applying the Smoluchowski equation:

*ζ* = 4*πημ*/*ε* (2)

(*ζ*—zeta potential (mV); *η*—viscosity; *μ*—electrophoretic mobility, *ε*—solvent’s dielectric constant).

The size and size distribution measurements were triplicated per run and were averaged from three independent runs. The zeta potential measurements were also triplicated per run and averaged from twenty runs.

### 2.7. Copolymer Micelles Preparation

The formation of nanosized micelles through a self-assembly of the amphiphilic triblock copolymers was achieved as follows: the respective copolymer was dissolved in a volatile organic solvent (acetone) and the concentration was adjusted to 10 mg mL^−1^. Afterwards, the copolymer solution (0.5 mL) was added slowly via a syringe to ~4 mL of vigorously stirred (900 rpm) water (ultrapure grade, 18.2 MΩ cm, pH 6.5). The acetone was easily removed from the mixture using a rotary evaporator and the formed aqueous dispersion concentration was adjusted to 1 mg mL^−1^ by the addition of ultrapure water. The micellar dispersions were filtered (0.45 μm pore size) and subjected to characterization.

### 2.8. Evaluation of the Critical Micelle Concentration (CMC)

The onset of the triblock copolymers’ self-association was evaluated in an aqueous milieu using the solubilization of a hydrophobic dye upon the micelles’ formation [51]. Briefly, a series of block copolymer solutions with gradually increasing concentrations (from 0.001 to 2.0 mg mL^−1^) were prepared followed by the addition of 10 μL from 0.4 mM solution in methanol of 1,6-diphenyl-1,3,5-hexatriene (DPH). After 18 h of incubation in the dark, the samples were subjected to UV spectroscopic analysis. The absorption intensity of DPH at λ_max_ = 356 nm was plotted as a function of copolymer concentration. The CMC values for the different triblock copolymers were obtained as the intersection point of the two straight lines from the absorption intensity vs. concentration plots.

### 2.9. Drug Loading Procedure and In Vitro Drug Release Profiles

The curcumin loading procedure for the triblock copolymer micelles was similar to that of the empty micelles. Initially, a Curc solution in acetone, with concentration 1 mg mL^−1,^ was prepared. In the next step, the triblock copolymer (10 mg) was dissolved into 1 mL from the drug solution. Afterwards, 0.5 mL from the solution containing both Curc and the triblock copolymer was slowly added via a syringe to ~4 mL of vigorously stirred (900 rpm) ultrapure water, followed by the organic solvent elimination and concentration adjustment to 1 mL^−1^. The drug-loaded nanocarriers (micelles to curcumin ratio–10:1 *w*/*w*) were passed through 0.45 μm syringe filter to remove the unloaded Curc and were recovered via lyophilization. The dried micelles were weighed, redissolved in acetone, and subjected to UV−Vis spectroscopic analysis. The extinction coefficient value, ε = 61,882 M^−1^ cm^−1^ (λ_max_ = 418 nm), of Curc obtained from a calibration curve in acetone was applied to calculate the amount of encapsulated into the nanocarriers’ drug. The drug loading efficiency (DLE) and drug loading capacity (DLC) were calculated by applying Equations (3) and (4):

DLE (wt%) = (the mass of encapsulated Curc /the input mass of Curc) × 100(3)

DLC (wt%) = (the mass of encapsulated Curc/total mass of the micelles) × 100(4)

The in vitro release profile of Curc was followed by applying the biphasic dissolution model. Typically, 6 mL of the aqueous Curc-loaded micelles’ dispersion (0.5 mg mL^−1^) were thermostated in vials at 37 °C. Then, 3 mL from the release media (chloroform) were added. At defined time intervals, 1 mL from the organic layer was withdrawn via syringe and analysed by UV/Vis spectroscopy (λ_max_ 415 nm, ε = 53 703 M ^−1^ cm^−1^). After each sample withdrawal, 1 mL of chloroform was added in order to keep the release media volume constant.

### 2.10. In Vitro Stability and Protein Adsorption

The in vitro stability and protein adsorption of Curc-loaded nanoparticles were investigated in phosphate-buffered saline (PBS) and in fetal bovine serum (FBS) solutions. Typically, 1 mL of PBS (pH 7.4) or 10% (*v*/*v*) FBS were added to equal volumes of nanocarrier dispersions (1 mg mL^−1^). The dispersions were gently mixed at 37 °C and the changes in the average particle diameters were followed by DLS measurements at predetermined time intervals (0, 3, 6, 24, and 48 h).

## 3. Results and Discussion

### 3.1. Synthesis and Characterization of Amphiphilic PLA-b-PDMAEMA-b-POEGMA Triblock Copolymers

**Scheme 1:**
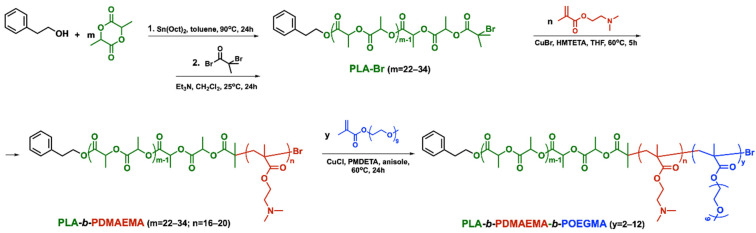
Synthetic steps towards amphiphilic PLA-b-PDMAEMA-b-POEGMA triblock copolymers.

The three-step synthetic procedure for the preparation of well-defined amphiphilic triblock copolymers with a tunable composition is presented in Scheme 1.

**Figure 1:**
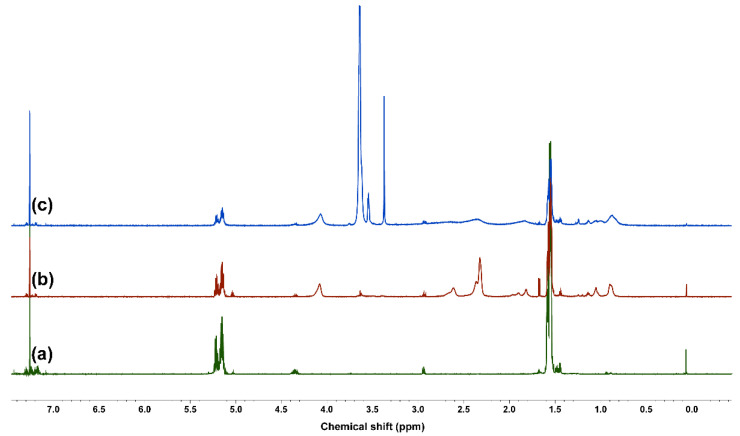
^1^H NMR (600 MHz) spectra in CDCl_3_ of: (**a**) hydrophobic biodegradable poly(D,L-lactide) (PLA, *DP*_n_ = 22); (**b**) amphiphilic PLA-b-PDMAEMA diblock copolymer (*DP*_n DMAEMA_ = 16, D2); (**c**) amphiphilic triblock copolymer with short oxyethylene side groups (PLA-b-PDMAEMA-b-POEGMA, *DP*_n OEGMA_ = 12, T2-3).

**Figure 2:**
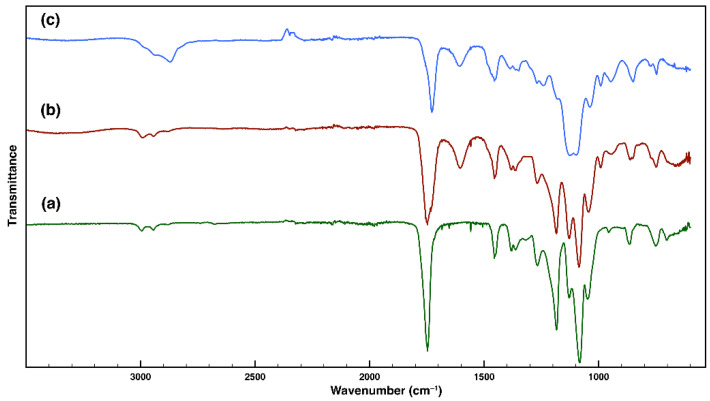
FTIR spectra of: (**a**) poly(D,L-lactide) (PLA, *DP*_n_ = 22); (**b**) the corresponding amphiphilic PLA-b-PDMAEMA diblock copolymer (D2); (**c**) amphiphilic triblock PLA-b-PDMAEMA-b-POEGMA copolymer (T2-3).

Initially, the hydrophobic poly(D,L-lactide) was synthesized via ring-opening polymerization of the corresponding cyclic lactide monomer, applying a modified literature procedure [47]. The polymerization was initiated by phenethyl alcohol, a functional initiator with easily detectable aromatic protons in NMR, and Sn(Oct)_2_ was used as a catalyst. The polymerization was performed in solution at an elevated temperature for 24 h. After the product purification via extraction, it was subjected to characterization. The PLA number for average molar mass was calculated from the ratio of the integral intensities of aromatic protons of the initiator at 7.30–7.15 ppm and methine protons of LA-repeating units of the polymer at 5.21–5.15 ppm (Figure 1a). Furthermore, the strong bands at 1082, 1184, and 1748 cm^−1^, corresponding to C-O-C (symmetric and asymmetric) and C=O stretching vibrations that are characteristic for the polyesters, are clearly visible in the FTIR spectrum of the product (Figure 2a).

**Figure 3:**
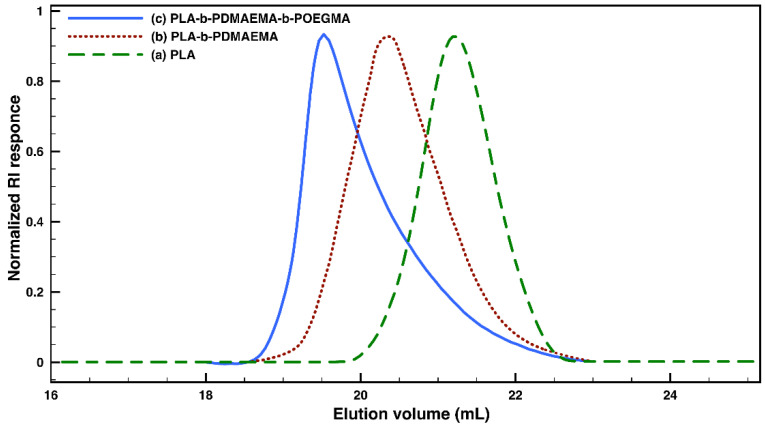
SEC elugrams of: (**a**) PLA-macroinitiator (M2, *Ð* = 1.15); (**b**) the corresponding amphiphilic PLA-b-PDMAEMA diblock copolymer (D2, *Ð* = 1.22); (**c**) amphiphilic PLA-b-PDMAEMA-b-POEGMA triblock copolymer (T2-3, *Ð* = 1.38) in tetrahydrofuran (*vs.* narrow PS standards).

SEC analyses demonstrated the formation of polymer with monomodal molar mass distribution and narrow dispersity (Figure 3a). The hydroxyl end-functionality of PLA was reacted with BIBB by applying a previously described procedure, thus converting the polymer into a macroinitiator for atom-transfer radical polymerization (ATRP) [48]. It was used in the second synthetic step to initiate the controlled polymerization of a methacrylic DMAEMA monomer (Scheme 1). The polymerization was performed in an oxygen-free atmosphere in THF for 5 h in the presence of a CuBr/HMTETA catalytic complex [49]. The purified product was characterized via ^1^H NMR analysis. With the knowledge of the macroinitiator’s degree of polymerization, the average length of the polycationic block was calculated as a ratio of the integral intensities of methylene protons of DMAEMA-units at 4.03 ppm and PLA methine protons at 5.21–5.15 ppm (Figure 1b).

The second block polymerization was also demonstrated by FTIR spectroscopic analysis of the purified product. (Figure 2b). The intense and characteristic bands that appeared at 1747 and 1732 cm^−1^ indicate the presence of carbonyl groups from both the PLA and PDMAEMA blocks. The bands at 1184 and 1085 cm^−1^, on the other hand, were assigned to the asymmetric and symmetric stretching vibrations of C-O-C functional groups.

The formation of diblock architecture was confirmed by SEC analysis. The elugrams of the diblock copolymers reveal a distinctive peak shift toward lower elution volumes corresponding to higher molar masses, whereas the molar-mass distribution remains monomodal with a slightly increased but still narrow dispersity (Figure 3b).

In the third synthetic stage, the inherent halogen end-functionality of the PLA-b-PDMAEMA diblock copolymer was used to initiate the ATRP of OEGMA-macromonomer (Scheme 1). The polymerization proceeded in anisole for 24 h in the presence of CuCl/PMDETA catalytic complex under modified conditions from the literature [50]. The product was purified via ultrafiltration in water and the degree of OEGMA polymerization was determined from the relative intensities of oxyethylene protons at 3.65 ppm and the methylene protons of DMAEMA repeating units at 4.03 ppm (Figure 1c). An additional strong band at 1100 cm^−1^ appeared in the FTIR spectrum of the product, corresponding to the vibrations of ether functional groups from the oligoethylene glycol side chains. (Figure 2c). The SEC-elugram of the final product shows a further shift toward higher molar mass and slightly higher dispersity when compared to the diblock precursor, depending on the degree of OEGMA polymerization (Figure 3c). The molar mass characteristics of the precursors and the triblock copolymers obtained are listed in Table 1.

### 3.2. Triblock Copolymers Self-Assembly

Because of their amphiphilic character, the synthesized polymers are prone to self-assembly in aqueous media. The nanoprecipitation technique was applied for the polymer micelles’ formation. Thus, a predetermined amount of the triblock copolymers was dissolved in an organic solvent (acetone), which is capable of dissolving all the copolymer segments. Moreover, it is miscible with water and can be easily removed from the mixture. The copolymer solutions were slowly added to a stirred water media, and, after the acetone elimination, the concentration of the formed micelles was adjusted to be 1 mg mL^−1^.

The micelles’ characterization is an essential step to be performed following their preparation. The critical micelle concentration (CMC) is a fundamental parameter used to characterize the thermodynamic stability of the micelles. Lower CMC values indicate greater thermodynamic stability. The values defining the micellization onset of the newly synthesized amphiphilic triblock copolymers were estimated by applying a hydrophobic dye signal spectroscopic detection technique, due to its ability to operate at very low polymer concentrations [51]. The appearance and the following changes in the UV-absorbance of DPH as result of its solubilization into the forming micelles was monitored as a function of the triblock copolymer concentration (Figure S1). The onset of micelles’ formation for the copolymers was estimated to be in the 0.07–0.24 mg mL^−1^ (7.5–20.5 μM) concentration range. The obtained data are in a good agreement with the copolymers’ hydrophilic–lipophilic balance, which was estimated according to the empirical model proposed by Griffin (Table 2) [52,53]. The CMC of the amphiphilic polymer micelles can be lowered by adjusting the length of the hydrophobic segment. Thus, the lowest CMC value of 0.069 mg mL^−1^ (hence micelles exhibiting the best thermodynamic stability) is demonstrated by the triblock copolymer T1, characterized by a longer hydrophobic block and the lowest HLB value of 9.07. For further characterizations in aqueous media, the concentration used for micelle dispersions preparation was 1 mg mL^−1^, a value significantly exceeding the determined onset of the micellization for all copolymers studied.

The DLS measurements were run both on the diblock and the corresponding triblock copolymer aqueous dispersions. In both cases, the instrument detected particles with sizes in the nanometer scale and relatively narrow size distributions (Table 2). The precursors self-assembled into micelles with diameters of 52 and 60 nm, whereas the triblock copolymer micelles formed micelles with sizes in the 65–72 nm range. The average micelles’ diameter increased after the polymerization of the third hydrophilic block (Figure S2).

**Figure 4:**
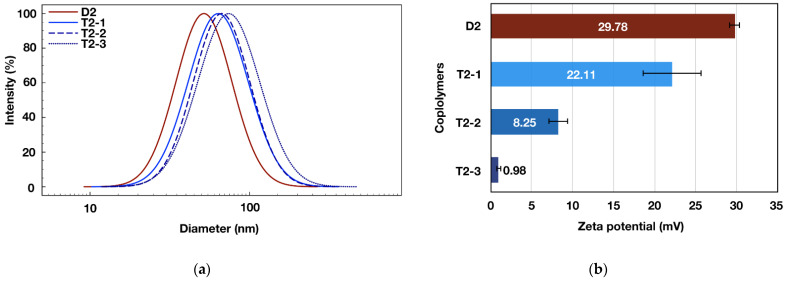
Micelles’ size distributions (**a**), and zeta potentials (**b**) obtained from dynamic light scattering analysis of aqueous dispersions (1 mg mL^−1^) of the diblock copolymer precursor D2 (d = 52 nm, PdI: 0.180, ζ = 29.78 mV) and the corresponding amphiphilic triblock copolymers: T2-1(d = 65 nm, PdI: 0.198, ζ = 22.11 mV), T2-2 (d = 67 nm, PdI: 0.193, ζ = 8.25 mV), T2-3 (d = 72 nm, PdI: 0.200, ζ = 0.98 mV).

Moreover, the size increased gradually with the degree of polymerization of the third block initiated by the same diblock copolymer (D2), indicating the formation of a thicker POEGMA outer shell (Figure 4a). Furthermore, the formation of a POEGMA shell with an increasing thickness is demonstrated by the micelles’ surface charge measurements (Figure 4b). The triblock copolymer micelles’ zeta potentials decreased gradually from strongly positive values (close to those of the diblock copolymer precursor) for the copolymers containing only a few OEGMA-units, to a completely shielded surface charge for the copolymer containing a longer POEGMA block.

Thus, by applying controlled polymerization techniques it is possible to control the degree of the triblock copolymer micelles’ surface charge. This, together with the optimal sub-100 nm average diameters of the particles obtained could be advantageous regarding their possible application in nanomedicine. It has been already shown that nanoparticles with similar sizes achieve an optimal cellular uptake as a result of the EPR effect when compared to smaller or bigger particles of the same nature [54].

**Figure 5:**
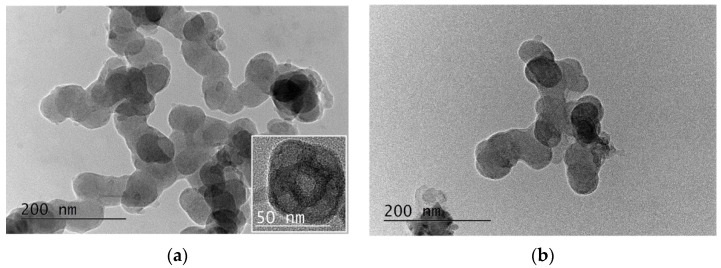
TEM micrographs of: (**a**) empty micelles (d = 55.93 ± 5.68 nm); (**b**) curcumin-loaded micelles (d = 63.32 ± 4.75 nm), obtained from the amphiphilic triblock copolymer T2-1.

Transmission electron microscopy was used to provide useful information on the structure/morphology of the formed micelles (Figure 5). The images revealed the presence of clusters of aggregated nanoparticles with a spherical shape. The measured average diameters are somewhat smaller but still in good correlation with the DLS results, taking into consideration the different conditions of the specimen that each technique requires. The visualized smaller particles by TEM are due to their shrinkage upon drying during the sample preparation. The shape of the individual copolymer micelle can be seen more clearly on the inset of Figure 5a.

### 3.3. Curcumin Loading into the Copolymer Micelles

The triblock copolymer micelles of different composition were loaded with the natural anticancer drug curcumin (Curc). The procedure was similar to that applied for the micellization. The only difference is that predetermined amounts from both the triblock copolymer and Curc were initially dissolved in acetone, forming a common solution. The organic solution was added slowly to ultrapure water upon stirring and, after acetone elimination, the dispersion concentration was adjusted by adding ultrapure water. The unloaded drug was separated from the dispersion by filtration. After lyophilization, the micelles were destroyed via dissolving them into acetone and the DLE and DLC were calculated according to Equations (3) and (4). The amount of the loaded curcumin into the micellar core was determined spectrophotometrically using a standard curve obtained by measuring the UV-absorption of different Curc concentrations in acetone (Figure S3). The calculated values for the drug loading efficiency ranged from 60 to 75%, depending on the triblock copolymer composition. The highest DLE value was obtained for the loaded nanocarriers formed from the triblock copolymer containing a longer PLA-block (T1) and a larger hydrophobic core. The calculated values for DLC were 7.3, 5.9, 6.5, and 6.1% for T1, T2-1, T2-2, and T2-3, respectively. The Curc-loaded polymeric nanocarriers were subjected to DLS analysis as well. The results indicate a slight increase in their average sizes, most likely due to the hydrophobic core expansion as a result of drug encapsulation. TEM analysis of the loaded copolymer micelles confirmed that there was no change in particles’ morphology as a result of curcumin encapsulation, just a slight increase of the average diameters (Figure 5b). Furthermore, there were no statistical changes in the measured zeta potentials of the drug-loaded micelles (not shown) when compared to their empty analogues, indicating that curcumin was located mainly within the nanocarriers’ hydrophobic core. The characteristics of triblock copolymer micelles before and after the drug loading are summarized in Table 2.

### 3.4. In Vitro Drug Release and Stability Measurements

The in vitro dissolution tests were performed in a hydrophobic organic solvent (chloroform) as a release media. Although well known, the so-called biphasic dissolution model for drug release monitoring is not frequently used, due to the existing possibility of micelles’ partial destruction at the water/organic solvent interface [55,56]. However, our attempts to perform in vitro drug release evaluations in PBS (pH = 7.4) as a release media using dialysis membrane and additives (different wt.% of ethanol or Tween^®^ 80) failed, due to adsorption of Curc onto the membrane material. Thus, in order to achieve comparable results, we applied the above-described biphasic method.

**Figure 6:**
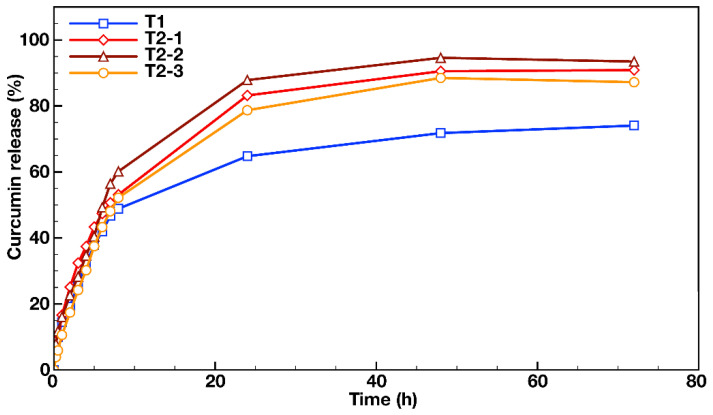
In vitro release profiles of curcumin from the different triblock copolymer micelles in a biphasic (water/chloroform) system at 37 °C.

The release media analysis of the series of triblock copolymer micelles with the same hydrophobic core and same middle PDMAEMA layer (T2-1, T2-2, and T2-3) revealed the achievement of a plateau for the released Curc, with 88–93% released drug after 72 h (Figure 6). The profiles were similar and two stages of Curc release was observed. Within the first eight hours, a rapid drug release took place, followed by a sustained release over the next hours. The effect of the initial rapid release was, however, more pronounced for the Curc encapsulated into the core of the nanocarriers formed from T2-2 copolymers than those of the T2-1- and T2-3-loaded micelles. In particular, 53 and 52% of Curc were released for 8 h from T2-1 and T2-3 micelles, respectively, while the initially released drug from T2-2 micelles was 60%. The higher initial burst release from T2-2 nanocarriers might be connected to their higher drug-loading efficiency relative to the other two analogues (Table 2). The release profile of Curc-loaded micelles of the amphiphilic triblock copolymer T1, characterized by the longer hydrophobic block (bigger micelle core), on the other hand, revealed a further decrease in the initial burst rate (48% for 8 h), despite the highest estimated drug-loading efficiency. This could be attributed to the enhanced stability of the nanocarrier (see CMC values in Table 2).

Furthermore, the incorporation of a significant amount from the hydrophobic drug into the micelle core could lead to a further stabilization of the structure. As a result, an overall of 74% of Curc was released after 72 h under the biphasic system conditions, which is much less than that achieved by the series of micelles obtained from the triblock copolymers T2-1, T2-2, and T2-3, characterized by smaller hydrophobic cores and higher HLB. Thus, by properly tuning the triblock copolymers’ composition, it is possible to achieve the desired balance between the contradicting requirements for the nanocarriers, namely the needed stability during the cargo transport through the blood stream and the efficient drug release at the site of action.

**Figure 7:**
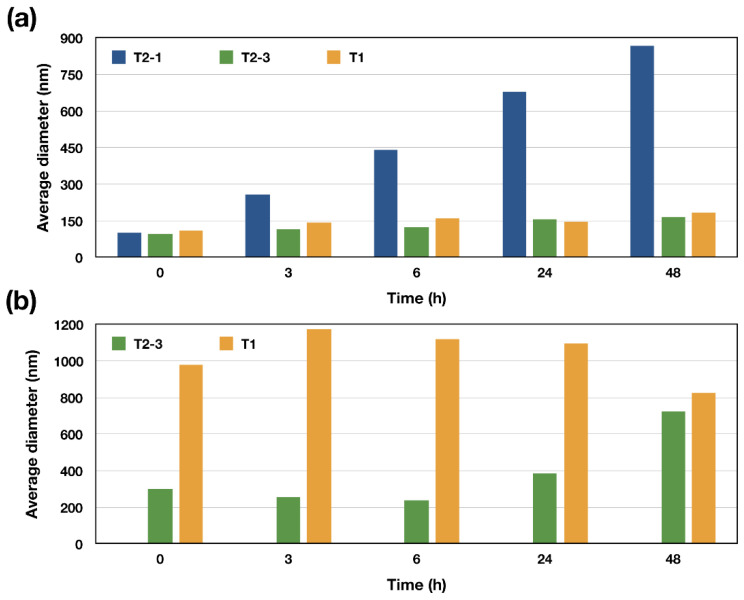
Stability of different triblock copolymer micelles assessed by DLS measurements in: (**a**) PBS buffer (pH 7.4); (**b**) FBS protein solution after various time intervals of incubation at 37 °C.

The nanocarriers’ stability was further evaluated mimicking the post-intravenous injection blood conditions, where the protein adsorption could result in micelles’ disassembly and unwanted drug release. Thus, selected drug-loaded micelles (from the least and the most POEGMA-functionalized triblock copolymers T2-1 and T2-3, as well as from the triblock copolymer T1 with the longer hydrophobic block) were incubated in phosphate-buffered saline, and DLS measurements were performed at predetermined time intervals to monitor the variations in micelles’ dimeters (Figure 7a). The results indicate that the loaded nanocarriers with a thick, non-charged corona (T2-3) and those comprising bigger hydrophobic core (T1) remain stable at physiological conditions during the period of evaluations. The Curc-loaded micelles with positively charged coronas and smaller hydrophobic cores (T2-1) started to increase their sizes on the third hour of evaluation, and on the 48th hour, they reached average diameters close to 900 nm, indicating a lack of stability in PBS. The same copolymer micelles showed a 20 nm increase of the average dimeters upon drug loading, which is also an indication of stability loss (Table 2). Therefore, for the following protein adsorption evaluation, only the Curc-loaded copolymer micelles of T1 and T2-3 copolymers were incubated in fetal bovine serum (FBS) solutions. The copolymer micelles with the thick POEGMA corona (T2-3) showed minimal fluctuations in their sizes up to the sixth hour of incubation in FBS, whereas the Curc-loaded micelles with a positively charged corona (T1) demonstrated a significant size increase from the initial moment of incubation (Figure 7b). Since the isoelectric point of FBS is lower than the experimental pH, the protein is negatively charged and immediately adsorbs onto the oppositely charged surface of T1-micelles. The fluctuations in both particle sizes could be attributed to protein adsorption and desorption processes on the micelles’ surface during the period of incubation.

After the 24th hour, even the T2-3 copolymer micelles dramatically increased their sizes due to FBS adsorption. However, it was demonstrated that the maximum micellar accumulation in the vicinity of the tumor site occurs within the 6 h after the injection [57]. Thus, as a result of series of controlled polymerizations and physico-chemical characterizations, it might be concluded that the micelles formed from the amphiphilic triblock copolymer, with densely grafted OEGMA units on the surface (T2-3), is a promising candidate for further in vitro and in vivo evaluations and for application in nanomedicine.

## 4. Conclusions

Amphiphilic triblock copolymers with predetermined compositions, comprising biodegradable poly(D,L-lactide), polycationic poly(N,N-dimethylaminoethyl methacrylate), and neutral poly(oligo(ethylene glycol) methyl ether methacrylate) hydrophilic blocks, were successfully synthesized by applying a three-step controlled polymerization procedure. The newly synthesized copolymers self-assembled in aqueous media into core–shell spherical micelles with average diameters of around 70 nm and tunable surface charges. They were used to encapsulate the natural hydrophobic model drug curcumin. The triblock copolymer micelles before and after the curcumin loading were physico-chemically characterized. It was demonstrated that parameters such as drug loading efficiency, drug release profiles, and micelles’ stability at physiological conditions can be modulated through the length of the copolymer’s hydrophobic block and the degree of particles’ surface charge. Moreover, the amphiphilic triblock copolymer with an optimal composition for the formation of stable nanocarriers of hydrophobic drugs with a potential application in nanomedicine was identified for further studies as an efficient drug delivery vehicle.

## Figures and Tables

**Table 1:** Molar mass characteristics of macroinitiators and the amphiphilic diblock and triblock copolymers.

Macroinitiators (PLA-Br)				Diblock Copolymers (PLA-b-PDMAEMA)				Triblock Copolymers (PLA-b-PDMAEMA-b-POEGMA)			
Code	*DP*_n_^a^ (LA)	*M*_n_^a^ (g mol^−1^)	*Ð*_M_^b^	Code	*DP*_n_^a^ (DMAEMA)	*M*_n_^a^ (g mol^−1^)	*Ð*_M_^b^	Code	*DP*_n_^a^ (OEGMA)	*M*_n_^a^ (g mol^−1^)	*Ð*_M_^b^
M1	34	5000	1.08	D1	20	8150	1.27	T1	2	9150	1.32
M2	22	3300	1.15	D2	16	5800	1.22	T2-1	2	6800	1.28
								T2-2	6	8800	1.33
								T2-3	12	11,800	1.38

^a^ Number of average molar masses (*M*_n_) and polymerization degrees (*DP*_n_), calculated from the ^1^H NMR spectra. ^b^ Molar mass dispersity (*Ð*_M_), estimated from the SEC analyses performed in tetrahydrofuran vs. narrow PS standards.

**Table 2:** Characteristics of triblock copolymer micelles before and after the drug loading.

	Empty Micelles					Drug Loaded Micelles			
Code	HLB ^a^	CMC ^b^ (mg mL^−1^)	d ^c^ (nm)	PdI ^c^	ζ ^c^ (mV)	d ^c^ (nm)	PdI ^c^	DLE ^d^ (%)	DLC ^d^ (%)
T1	9.07	0.069	72.26 ± 0.21	0.158	21.49 ± 4.17	74.14 ± 0.21	0.135	75	7.3
T2-1	10.29	0.088	65.42 ± 1.01	0.198	22.11 ± 3.60	82.88 ± 1.14	0.170	60	5.9
T2-2	12.50	0.129	66.83 ± 0.22	0.193	8.25 ± 1.25	71.46 ± 0.70	0.156	67	6.5
T2-3	14.41	0.243	71.73 ± 0.29	0.200	0.98 ± 0.34	73.94 ± 0.63	0.217	63	6.1

^a^ Hydrophilic–lipophilic balance (HLB) determined from the ratio between the molar mass of the hydrophilic segments and the overall copolymer molar mass, and multiplied by 20. ^b^ Critical micelle concentration determined spectroscopically. ^c^ Average micelles’ diameters (d), size distributions (PdI) and zeta potentials (ζ) obtained from DLS measurements. ^d^ Drug loading efficiency (DLE) and drug loading capacity (DLC) determined spectroscopically.

## Data Availability

Not applicable.

## Supplementary Materials

The following supporting information can be downloaded at: https://www.mdpi.com/article/10.3390/nano12030434/s1, Figure S1: Effect of block copolymer concentration on the absorption intensity of DPH at 356 nm in aqueous media for: (a) amphiphilic triblock copolymer T1 (HLB = 9.07); and (b) amphiphilic triblock copolymer T2-3 (HLB = 14.41). Figure S2: Size-distribution curves obtained from DLS measurements of 1 mg mL^−1^ aqueous micellar dispersions of the amphiphilic diblock copolymer precursor D1 (d = 60 nm, PdI: 0.154, ζ = 35.64 mV) and the corresponding triblock copolymer T1 (d = 72 nm, PdI: 0.158, ζ = 21.49 mV). Figure S3: Calibration curve constructed from the UV-absorption of different curcumin concentrations in acetone.
