# Supplementary material for: Triblock Copolymer Micelles with Tunable Surface Charge as Drug Nanocarriers: Synthesis and Physico-Chemical Characterization
Source: Nanomaterials (Basel). 2022 Jan 27;12(3):434. doi: 10.3390/nano12030434 (PMC8840746; doi:10.3390/nano12030434)
Supplement: Supplementary file 1 [file nanomaterials-12-00434-s001.zip › nanomaterials-1552475-SI-done/nanomaterials-1552475-SI-done.pdf]

nanomaterials

Supplementary Materials

Triblock Copolymer Micelles with Tunable Surface Charge as Drug Nanocarriers: Synthesis and Physico-Chemical Characterization

Radostina Kalinova and Ivaylo Dimitrov *

Institute of Polymers, Bulgarian Academy of Sciences, Akad. G. Bonchev St., bl. 103-A, 1113 Sofia, Bulgaria; kalinova@polymer.bas.bg
* Correspondence: dimitrov@polymer.bas.bg; Tel.: +359-2979-3628

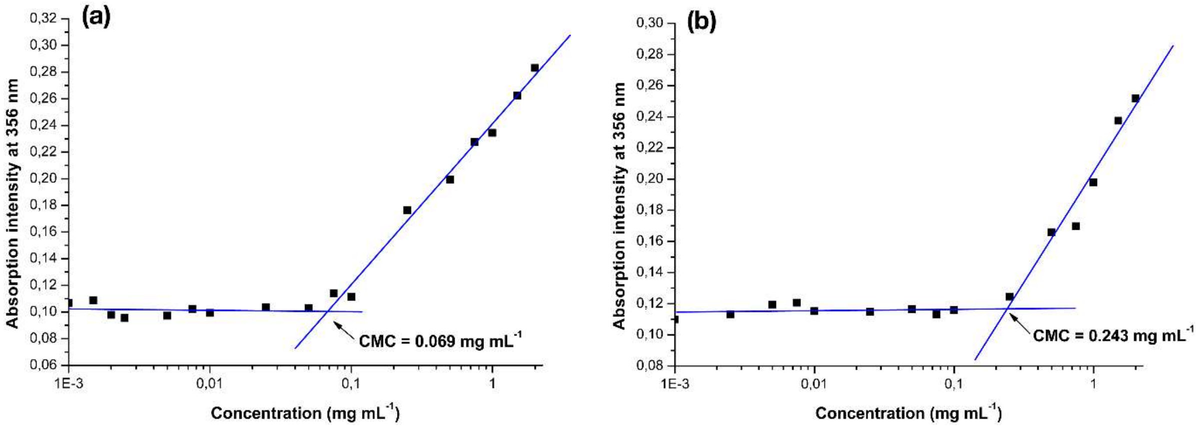

Figure S1. Effect of block copolymer concentration on the absorption intensity of DPH at 356 nm in aqueous media for: (a) amphiphilic triblock copolymer T1 (HLB = 9.07); and (b) amphiphilic triblock copolymer T2-3 (HLB = 14.41).

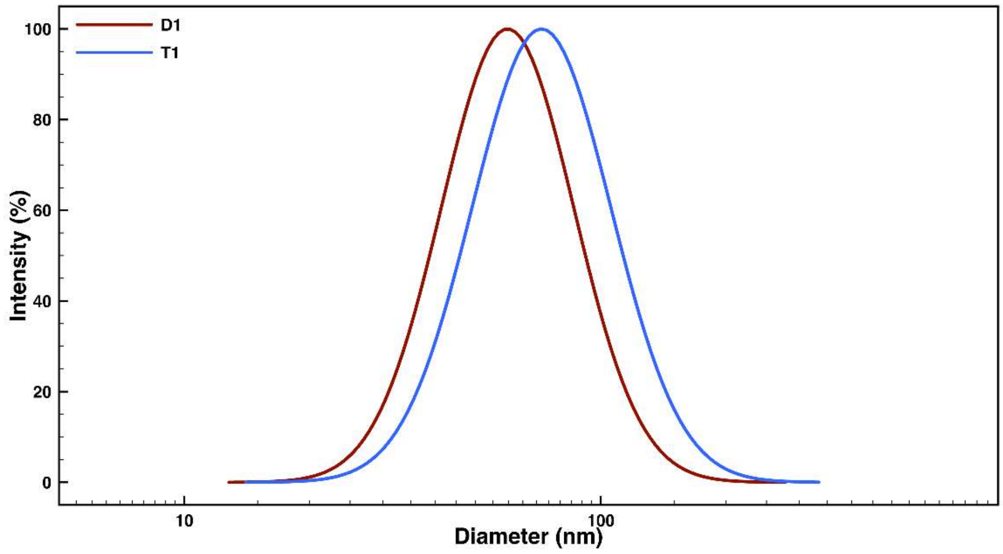

Figure S2. Size-distribution curves obtained from DLS measurements of 1 mg mL^{-1} aqueous micellar dispersions of the amphiphilic diblock copolymer precursor D1 (d = 60 nm, PdI: 0.154, ζ = 35.64 mV) and the corresponding triblock copolymer T1(d = 72 nm, PdI: 0.158, ζ = 21.49 mV).

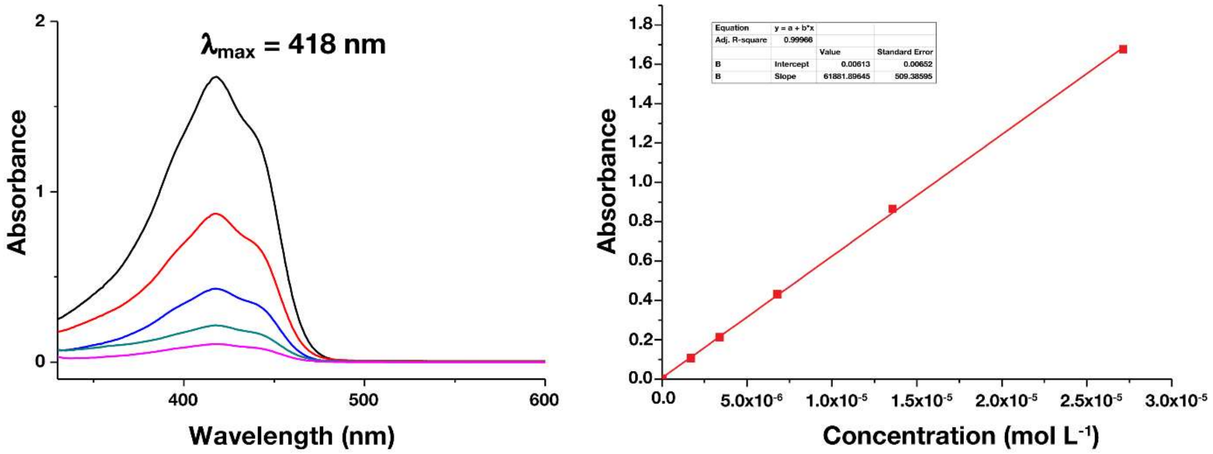

Figure S3. Calibration curve constructed from the UV-absorption of different curcumin concentrations in acetone (λ_{max} = 418 nm, ε = 61 882 M^{-1} cm^{-1}).
